# An Overview of Donkey Neonatology

**DOI:** 10.3390/ani15131986

**Published:** 2025-07-06

**Authors:** Francisco J. Mendoza, Ramiro E. Toribio

**Affiliations:** 1 Department of Animal Medicine and Surgery, Veterinary Faculty, University of Cordoba, 14014 Cordoba, Spain; 2 Department of Veterinary Clinical Sciences, College of Veterinary Medicine, The Ohio State University, Columbus, OH 43210, USA; toribio.1@osu.edu

**Keywords:** diarrhea, gastrointestinal disease, neonatal maladjustment syndrome, newborn and neonate donkey, sepsis, umbilical disorders

## Abstract

The number of donkey-breeding farms is variable around the world despite the growing popularity of this species as working animals, companion pets, for show events, in asinotherapy for children and adults with physical and mental disorders, and as a source of high-quality nutritional products. Despite this, due to the low foaling rate, long foaling interval, and high neonatal mortality rate, the number of donkey foals every year has been decreasing. This is a major problem for breeds in danger of extinction, where each foal is highly valuable. Although there is extensive information on equine foals, data on donkey foals is scarce. Donkey foals are afflicted by the same disorders as equine foals, with sepsis, failure of transfer of passive immunity, dysmaturity, and umbilical disorders being the most common ones. The diagnosis and therapeutic approaches for these conditions are similar to equine foals, but inter-species differences should be taken into consideration. Therefore, there is an increasing need for studies on healthy and sick donkey neonates, because this information will not only increase our understanding of their physiology but it will also impact the prevention and treatment of conditions affecting these animals, therefore improving survival rates.

## Simple Summary

Donkey popularity has been steadily increasing around the world, resulting in a higher need for specialized veterinarians with specific knowledge of this species. Although information about donkeys is scarce, even less is known about donkey neonatology compared to horses. Disorders affecting equine foals, such as sepsis, neonatal encephalopathy, failure of transfer of passive immunity, and dysmaturity, also occur in donkey foals. Anatomical, physiological, pharmacological, metabolic, and endocrine differences compared to horses have been documented in donkeys and should be considered in the evaluation, diagnosis, prognosis, and treatment of disorders of donkey neonates.

## 1. Introduction

Scarce information on healthy and sick donkey foals compared to equine foals is available, limiting our understanding and subsequent approach to some perinatal disorders [1]. Due to this limited information, clinicians extrapolate data from equine foals, with the subsequent risk of misdiagnosis, inappropriate drug use, potential side effects, and unnecessary expenses. Since important differences have been previously reported between adult donkeys and horses [2,3,4], we can assume that many of these differences already exist in the neonatal period.

Depending on the region, mostly in low-income countries, mortality in donkey foals is higher than in horse foals, mainly due to minimal veterinary care, poor hygienic conditions, unsupervised foaling and pregnancy (higher twin pregnancies compared to mares), and financial limitations [5]. Considering that jennies have lower foaling rates (approximately 75%), longer gestation periods (average 370 days), and longer foaling intervals (average 500 days) than mares [6,7] and that the worldwide donkey population has been declining, the survival of each donkey neonate deserves additional attention, particularly local donkey breeds that are close to extinction. In addition, it is important to emphasize countries with the highest number of donkeys (China, Middle East, and Africa) are also those with financial limitations, making veterinary care and preventive practices even more challenging, further favoring the decrease in the worldwide donkey population. Although some countries and organizations have been promoting the conservation of endangered donkey breeds and owner education, the economic situation must be taken into consideration.

This review provides information on donkey neonatology, including known differences with equine foals and species-specific clinical considerations that could increase the survival rate of these foals.

## 2. General Considerations of Healthy Donkey Foals

### 2.1. Physical Examination of Healthy Donkey Foals

The age grouping previously described for equine foals could also apply to donkey foals, with a newborn donkey foal considered from birth through 3 days of age, a neonate donkey foal from 4 to 14 days of age, a juvenile foal from 15 days to 6 months of age, and a weanling foal from 7 months to 1 year of age [8].

The evaluation of donkey foals is not different from equine foals. After birth, the donkey foal should be standing within one hour, nursing and passing meconium should occur within two hours, and the jenny should expel the fetal membranes by three hours (usually earlier than two h) [9]. Similar to other species, maternal contact with the neonate stimulates oxytocin release, milk let down, and uterine contractions, which favor the expulsion of the fetal membranes. The time to first urination is similar to equine foals, usually within 9 h, with no sex differences [10].

A complete physical evaluation should be performed on newborn donkeys, ideally within 24 h. The process is similar to equine foals, considering their unique differences. This should include an evaluation of cardiovascular function, heart rate, and respiratory rate; palpation of the rib cage and appendicular apparatus; abdominal auscultation; and an ophthalmic assessment. It is also important to evaluate the donkey foal while it is standing and walking to observe potential orthopedic problems, like lameness, as well as flexural and angular deformities. Heart and respiratory rates are not different than equine foals [10]. The rectal temperature is 37–38.5 °C (98.6–101.3 °F). Despite that donkey foals have a denser haircoat, they are more prone to hypothermia than equine foals, regardless of breed, management, and health status. Therefore, it is important to protect them from cold conditions [11]. External umbilical structures should be evaluated for early detection of abnormalities, such as patent urachus (wet navel), and infections (exudate, pain, and warm). Umbilical dipping in 0.5–2% chlorhexidine or 1% povidone iodide is recommended for topical disinfection. In addition, it is important to keep the umbilical area dry. Excessive manipulation or umbilical ligation predisposes it to swelling and complications, such as omphalophlebitis [12].

Equine neonates must consume between 2–4% of their body weight in good quality colostrum (Brix value > 14% or specific gravity > 1060) to achieve adequate IgG concentrations before of 12 h of life. In the first week of age, healthy donkey foals nurse 5–6 times/h (1–2 min each time), and it is estimated that they consume 20% of their body weight. In orphan donkey foals, a milk replacer is a good alternative or a starter grain at 2% of the foal’s body weight from the first 24 h of life can be used [13,14]. The daily weight gain depends on the breed. For example, in some breeds, such as Martina Franca, it has been estimated to be 0.5 kg/day [15], while in most breeds (including miniature), it is less. Similar to horses, donkey foals are weaned at 5–6 months old, and they can start to consume forage at an early age (approximately 2 months old) [14].

Every donkey must be closely evaluated during the first 24–48 h after foaling, especially those born from high-risk jennies or from complicated deliveries. A complete assessment should include a complete physical evaluation and systemic assessment through hematology, serum biochemistry, and the measurement of IgG concentrations. The Apgar score has been characterized in donkey foals [10]. This score is based on heart and respiratory rates, muscle tone, mucous membrane color, and irritability reflex in order to assess foal viability after birth (Table 1). Donkey foals with Apgar scores lower than 6 out of 8 points have a higher risk of developing complications [10].

### 2.2. Laboratory Findings in Healthy Donkey Foals

Since hematologic and biochemical differences have been reported between adult donkeys and horses [4,17], it is paramount to consider that they are also observed between donkey and equine foals. Moreover, age and breed should also be taken into consideration in order to interpret blood analysis results.

#### 2.2.1. Hematology

Red cell parameters, for equine foals, decrease in the first week of life to values close to adults (Table 2). These changes could be due to an increased erythrocyte destruction, reduced production from changes in oxygen tension, or a dilutional effect. The hematocrit and hemoglobin concentrations are higher in donkey foals than in equine foals [18,19,20].

No significant differences in white blood cells (WBCs) have been documented between equine and donkey foals (Table 2) [18,19,20], although some studies have shown minor differences, with lower neutrophil and monocyte counts but higher lymphocyte, eosinophil, and basophil counts compared to equine foals of similar age [18,19,20]. Similar to equine foals, the WBC increases in the first 4 days after birth and reaches adult values by 1 week of age [19].

The platelet count and platelet indices change minimally in the first week of age, and differences observed between both species are minimal, and they have no clinical relevance [18,19,20].

#### 2.2.2. Biochemistry

Serum glucose concentrations in donkey foals are higher than in horse foals at birth and are higher than in adult donkeys (Table 3) [19,20]. Serum triglyceride concentrations increase from birth to the first week of life [19], similar to equine foals [22]. The feeding milk replacer for donkey foals can increase serum triglyceride concentrations without changes in plasma glucose concentrations [14].

The serum total protein and albumin concentrations remain unchanged in the first week of life, and values are similar to equine foals (Table 3) [19,20]. Plasma IgG concentrations are similar between donkey and equine foals at birth, reaching maximum concentrations at 24 h [24,25].

Similar to equine foals [26], serum blood urea nitrogen and creatinine concentrations are higher at birth and steadily decrease by 48–72 h to normal values, which should not be confused with spurious azotemia due to placental insufficiency [19,20].

Similar to equine foals [27,28], blood L-lactate concentrations are high at birth and drop to normal concentrations by 24–48 h of age [20,23]. One study found higher L-lactate concentrations at birth in donkey foals compared to equine foals [20].

An increase in aspartate aminotransferase (AST) activity in the first 3 days of life has been observed, likely from muscle activity, although creatine kinase (CK) activity changes minimally or decreases [19,20]. Alkaline phosphatase (ALP) activity is high at birth and decreases with aging [26], although inter-breed differences have been observed [19]. Gamma-glutamyl transferase (GGT) and lactate dehydrogenase (LDH) activities show minimal changes in the first week of life, but GGT activity may increase in the first 24 h in both species (Table 3) [19,29].

Serum sodium, chloride, potassium, calcium, and phosphorus concentrations are similar between donkey and equine foals immediately after birth (Table 4) [19,20,26]. Regarding blood gases, pH and arterial PCO_2_ and PO_2_ are similar between donkey and equine foals [20,23,30,31,32], but a lower venous PO_2_ in donkey than in equine foals has been reported [31,32]. HCO_3_ concentrations are similar to adult donkeys [20,30] and horse foals [31].

## 3. Considerations of Sick Donkey Foals

### 3.1. Sepsis

Sepsis, defined as a life-threatening condition involving a proven or suspected infection associated with systemic inflammation [33], is a major cause of co-morbidity and mortality in neonate donkey foals similar to equine foals. Although donkey-specific epidemiological data are not available, the prevalence of sepsis seems to be higher than in equine foals, likely due to minimal veterinary care, poor management, inadequate hygienic conditions, and scarce knowledge of early detection and interventions on disorders of the perinatal period in this species. The last point is valid for both developing and developed countries.

Similar to equine foals, in addition to IgG concentrations, environmental conditions and management are major predisposing factors for neonatal infections. Infections can occur in utero from jennies with placentitis but more often happen immediately after birth. Umbilical infections and the gastrointestinal tract are the main routes for bacterial entry and hematogenous dissemination. Entry can also occur from the respiratory tract. Foals born to dystocia, with maturity disorders or with other concomitant disturbances, such as failure of transfer of passive immunity (FTPI) or neonatal encephalopathy (NE)/neonatal maladjustment syndrome (NMS), have a higher risk for sepsis and should be monitored closely [21,34].

Clinical signs of sepsis are not different from equine foals [35], ranging from fever, lethargy, anorexia, weakness, reluctance to move, low interest by the mother, and recumbency to organ dysfunction [34]. Secondary clinical signs depend on affected organs and can include diarrhea, respiratory distress, patent urachus, a painful umbilical stump, uveitis, seizures, lameness, and joint swelling.

The diagnosis is made using clinical and laboratory findings as well as ancillary methods. Markers of systemic inflammatory response syndrome (SIRS), such as the rectal temperature, respiratory and heart rates, and WBC count, were recently shown not to be predictive of sepsis [36]. This information has not been studied in donkey foals, but it is likely similar. Inflammatory biomarkers, such as serum amyloid A (SAA) concentrations, the neutrophil:lymphocyte ratio, and the red cell distribution width-to-platelet ratio [37,38], have not been evaluated in donkey foals (both healthy and septic), although some information on SAA in adult donkeys is available [39].

A blood culture or a culture from suspicious infected sites must be performed in any equid neonate to identify the etiologic agent, for proper antimicrobial selection, and for prognosis [34]. No species-specific information is available in donkey foals, but the pathogens seem to be similar to those described for equine foals. The most common bacteria include *E. coli*, *Actinobacillus* spp., *Enterococcus* spp., *Enterobacter* spp., *Klebsiella* spp., *Pseudomonas* spp., *Streptococcus* spp., and *Staphylococcus* spp. [40]. Equine herpesvirus and equine viral arteritis can also cause serious diseases in newborn foals [34].

The sepsis score developed for equine foals can be used, but it has not been validated for donkey foals [41,42]. Foals with a score >12 (maximum score: 38) have >93% likelihood of sepsis [41]. The Apgar score can be useful for assessing the overall health of a newborn donkey foal [10], however, its clinical value in sick donkeys remains to be determined. An Apgar score <6 out of 8 indicates higher odds of complications and lower survival rates [10].

Other laboratory findings related to multiorgan involvement are azotemia, hypoglycemia, an increase in liver enzyme activities, hemoconcentration, hyperlactatemia, metabolic acidosis, and hypocalcemia.

The treatment of a septic donkey foal follows the same principles as equine foals [34], including antimicrobials (Table 5), anti-inflammatory drugs (Table 5), fluid therapy, enteral or parenteral nutrition, and the correction of acid–base and/or electrolyte imbalances. Intravenous antimicrobials must be administered as soon as possible, and the decision to discontinue should be based on clinical and laboratory improvements following the same principles applied to equine foals, including improvements on clinical findings, WBCs, fibrinogen, and SAA concentrations. A broad-spectrum antimicrobial combination with a β-lactam (ampicillin or penicillin) and an aminoglycoside (amikacin or gentamicin) is a good initial step until the results of the bacterial culture are available (Table 5). Aminoglycosides should be avoided in azotemic foals. Due to antimicrobial stewardship practices imposed in some countries, the use of third- or fourth-generation cephalosporins is restricted, at least until a bacterial culture with susceptibility is available (Table 5). Intravenous sulfonamides can also be administered, although they are available only in some countries (Table 5).

Similar to equine foals, additional medical treatment will depend on clinical findings and complications and may include plasma transfusion, furosemide, anti-ulcer drugs, misoprostol, oxygen, caffeine, bronchodilators, and vasopressors [34].

The survival rate of septic donkey foals is lower compared to equine foals for a number of reasons, including stoic behavior, the delayed identification of signs of sepsis, the lack of veterinary attention in developing countries, hygiene, poor environmental conditions, and financial limitations. Like in equine foals, additional disturbances, such as septic arthritis, enterocolitis, and umbilical disorders, decrease the survival rate [40].

### 3.2. Failure of Transfer of Passive Immunity (FTPI)

The prevalence of FTPI in donkey foals appears to be similar to equine foals, at approximately 30% [43]. Predisposing factors for FTPI are maternal (lack of udder development, premature lactation and colostrum loss, maternal diseases, maternal rejection (caution in maiden or feral jennies), and poor-quality colostrum), placental (placentitis and placental separation), and neonatal disorders (congenital or acquired perinatal disorders) that impair nursing and inadequate ingestion of colostrum (e.g., sepsis, dysmaturity, musculoskeletal disorders, etc.), gastrointestinal disease impairing absorption, and environmental conditions (management, hygiene, and infectious agents) [21].

FTPI does not have specific clinical signs, and its diagnosis is based on clinical history and serum IgG concentrations. Methods used to measure IgG concentrations in equine foals work well in donkey foals [25], both quantitative (e.g., single radial immunodiffusion (gold standard) and colorimetry) and semiquantitative (e.g., glutaraldehyde coagulation test, zinc sulfate turbidity test, ELISA (SNAP Foal test), and latex agglutination). Serum IgG should be measured after 12 h of age to obtain reliable values.

Information on the association between different IgG concentrations and disease severity in donkey foals is lacking. Thus, it is advisable to follow the FTPI categorization for equine foals: blood IgG concentrations >800 mg/dL are considered adequate for the transfer of passive immunity, 400–800 mg/dL as partial FTPI, and <400 mg/dL as total FTPI [35].

Therapeutic approaches will depend on whether the enteral route is viable and on foal age. In foals of less than <12 h of age with a functional gastrointestinal tract, administering good-quality colostrum through a nasogastric tube is a feasible and cheap option. Colostrum can be also administered in donkey foals younger than 6 h of age, if they were born from high-risk pregnancies, from jennies with known poor-quality colostrum, or from jennies with udder disorders. Similar to mares, colostrum quality can be estimated using colostometers or Brix refractometry. In mares, it has been established that a colostrum density >1060 is consistent with good quality colostrum, and this principle likely applies to jennies. For Brix refractometry, values of 14–17% and >17% were considered adequate or very good, respectively. In contrast, Brix values of 20–30% (50–80 g/L) and 30% (>80 g/L) in mares were previously considered adequate or very good, respectively [44]. If donkey colostrum is not available, equine colostrum works well in donkeys; however, it is important to monitor the donkey foals for a few days, because they may develop immune-mediated anemia (IMHA) and immune-mediated thrombocytopenia (IMTP). Commercial equine colostrum can also be used in donkey foals.

Depending on the size of the neonate, 100–300 mL of colostrum for a 25–45 kg donkey foal should be administered and repeated within a few h until a total of 4% of body weight (ideally before 12 h of age) to achieve adequate IgG concentrations.

If colostrum is not available, the foal is older than 18 h, or the foal suffers from gastrointestinal disorders, plasma transfusion must be performed. Plasma from the mother or a castrated donkey should be considered. If equine plasma is used, plasma from an equine gelding is preferred. If the donor is a mare, then mares with no history of having mule foals or foals with neonatal isoerythrolysis should be used. Commercial plasma is better, because it is free of pathogens, and donors have been tested against major equine incompatibility antigens, including the donkey factor. The amount of plasma ranges from 250–500 mL for miniature donkeys to 1–2 L for larger donkey foals (30–50 kg). If equine plasma is administered, serial hemograms should be performed for the next 48 h to detect immune-mediated disturbances, such as IMHA or IMTP [21].

### 3.3. Umbillical and Urogenital Disorders

The prevalence of urogenital problems in donkey foals remains to be reported; however, acquired and congenital conditions are observed frequently. Congenital conditions such as hypospadias and hydronephrosis have been documented [45].

The most common conditions include omphalitis, omphaloarteritis, omphalophlebitis, and patent urachus, which often lead to bacterial entry and sepsis as well as secondary complications (e.g., diarrhea, septic arthritis, physitis, pneumonia, and meningitis) due to hematogenous dissemination. Other conditions, such as umbilical vein or liver abscesses, uroabdomen, and renal failure, can also be seen [12].

Weak foals or those with musculoskeletal disturbances resulting in recumbency are more likely to develop umbilical infections [46]. Poor hygiene and inadequate environmental conditions are also predisposing factors for infections. Pain, heat, umbilical stump enlargement, urine dripping, or a moist navel are the most common signs [12]. An infection and rupture of the urachus predispose foals to uroabdomen.

Diagnosis is based on ultrasonography of umbilical structures using species-specifics values. The size of both the umbilical arteries and umbilical vein must be less than 0.8 cm, while the internal umbilical remnant should be less than 2.2 cm [47]. These values are slightly smaller for miniature donkey breeds. An enlargement of these structures, abscessation, or the presence of free fluid within the remnants are abnormal [12]. Anechoic or hypoechogenic free fluid in the abdominal cavity showing twice the creatinine concentration compared to serum is indicative of uroabdomen. In foals with uroabdomen, electrolyte imbalances are similar to equine foals (hyperkalemia, hyponatremia, and hypochloremia); thus, special consideration must be paid to fluid administration, and anesthesia should not be performed up to electrolyte imbalance correction [48].

The initial medical treatment should be based on broad-spectrum systemic antibiotics. Surgery is usually an elective (non-emergency) procedure and/or conducted when the response to the initial medical treatment alone is unsatisfactory [12]. Hyperkalemia is corrected following steps that are similar to those described for horse foals: free-potassium fluids, glucose, bicarbonate, calcium, and in severe cases, insulin [48].

Omphalectomy via a ventral midline laparotomy to remove umbilical structures is commonly performed in foals with umbilical disorders and should be considered in foals with secondary infections, such as septic arthritis, to prevent future umbilical disturbances and complications [12]. Surgical bladder repair follows a similar pattern as equine foals [12].

Renal dysfunction can be primary or secondary to infectious and non-infectious causes. The principles of pre-renal and renal azotemia apply to donkey foals. Dehydration signs are not well correlated with the degree of hypovolemia in donkeys; thus, if there is pre-renal azotemia, the water deficit is likely higher than that represented by clinical signs. Milk deprivation due to the jenny not having sufficient milk, the foal not having interest due to systemic disease (anorexia), or physical limitations can result in rapid dehydration. This is frequent in donkey foals with sepsis, prematurity, or maladjustment syndrome [21].

Like equine foals, donkey foals are born with high creatinine concentrations that decrease rapidly and reach normal values by days 2–3 of age [23]. Similarly, L-lactate concentrations are high at birth and drop to normal values by 2 days of age [23]. Any condition that reduces the blood volume or interferes with tissue perfusion or oxygenation (e.g., NMS, sepsis, pneumonia, anemia, and cardiac disease) will increase L-lactate concentrations.

The treatment of pre-renal and renal azotemia is similar to equine foals, restoring the blood volume and tissue perfusion [48]. Diuretics (furosemide) and vasopressors can also be administered to improve renal perfusion, increase the glomerular filtration, and urine output [48]. Caution must be taken when treating foals with azotemia or hyperlactatemia with nephrotoxic drugs.

### 3.4. Maturity Disorders

To our knowledge, epidemiological studies on disorders of maturity (prematurity, dysmaturity, and postmaturity) in donkeys are lacking, but the same concepts used for horses apply to this species [49]. It is important to know that jenny gestation is longer (mean: 370 days) compared to mares [6]. Postmaturity due to fescue toxicity has not been described in donkeys.

Although no specific information is available for donkey foals, predisposing factors for disorders of maturity described in horses could be extrapolated to donkey foals. These risk factors include maternal diseases (e.g., placentitis, hyperlipemia, etc.), placental dysfunction, fetal disorders (e.g., micromineral deficiency), congenital defects, and stress [49].

Signs of prematurity (shorter gestational time) or dysmaturity are similar to those described in equine foals, including weakness, the inability to stand, a domed head, floppy ears, fuzzy haircoat (for a donkey), delayed hoof cornification, lameness, tendon laxity, and respiratory distress [35].

The diagnostic approach for premature and dysmature donkey foals is not different than equine foals [49], and it should be mainly based on the physical appearance of the foal due to variability in gestation length in donkeys. Laboratory information for premature donkey foals is lacking, but it can be assumed to be similar to equine foals (low neutrophil-to-lymphocyte ratio). It is suggested to measure IgG concentrations because, unless they receive colostrum or plasma, they will have FTPI and will become septic. Radiographs of the carpus and tarsus are valuable to assess the degree of ossification of cuboidal bones. Thoracic radiographs are also important to assess pulmonary parenchyma and maturity (pulmonary atelectasis). Thoracic and abdominal ultrasonography is also recommended [21].

Due to the potential immune incompetent status of these immature animals and the risk of bacterial translocation, it is suggested to administer antimicrobials (therapeutic combinations similar to those previously described for sepsis) and plasma transfusion [21,49]. Other medical support recommendations are not much different than in equine neonates. Protection against hypothermia is crucial in premature and dysmature donkey foals due to their lower capacity for thermoregulation and predisposition to hypothermia compared to equine foals.

### 3.5. Neurological Disorders

Congenital (hydranencephaly and anencephaly) [50] and acquired neurological disorders can be observed in donkey foals (Table 6).

Observing the behavior of the jenny and the newborn donkey foal is important for identifying problems specific to the foal and the mother and also for bonding, which should be strong [51]. For example, the jenny rejecting the foal, the jenny showing aggressiveness, or frequent attempts to nurse by the foal could indicate a problem with the jenny. In contrast, donkey foals lying for too long or that are not interested in the mother could not only indicate an underlying systemic problem with the foal (e.g., NMS or sepsis) but could also be a problem with the jenny (e.g., not having sufficient milk or rejection).

No epidemiological data on neurological disturbances affecting donkey foals is available, although they could be higher than in equine foals [10]. Neonatal encephalopathy (NE) or neonatal maladjustment syndrome (NMS), also named hypoxic-ischemic encephalopathy (HIE) and perinatal asphyxia syndrome (PAS), is frequent in equine foals, although the prevalence in donkey foals is unknown. The risk factors for NE are likely similar to equine foals, including maternal illnesses leading to decreased uteroplacental perfusion (endotoxemia, hypovolemia, anemia, hypotension, and respiratory dysfunction), placental diseases (premature separation, insufficiency, placentitis, and umbilical cord torsion), parturition problems (dystocia, twins, and postmaturity), and neonatal issues (dystocia, congenital respiratory problems, respiratory insufficiency (prematurity), and meconium aspiration) [52]. In many instances, the underlying cause leading to NMS in donkey foals is unknown.

A specific diagnosis of NE (NMS) is not available, and diagnosis is reached by clinical history, clinical signs, and discarding other differential diagnoses [52]. Clinical signs can appear shortly after birth, or the foal can be normal for the first 12–24 h of life and then develop signs. The clinical signs are variable and are similar to equine foals, showing changes of consciousness (disorientation, lethargy, hyperexcitability, etc.), behavior (without an interest for the jenny, problems with udder seeking, wandering, etc.), and neurologic dysfunction (ataxia, an absent or poor suckling reflex, head pressing, the inability to stand, an abnormal respiratory rate or pattern, dysphagia, seizures, etc.) [52]. Secondary problems related to poor nursing, such as dehydration, hypoglycemia, FTPI, and sepsis, or those related to organ dysfunction secondary to hypoxia, such azotemia due to acute renal failure, enterocolitis, or arrhythmias, can also be observed.

Metabolic causes of encephalopathy, such as hypoglycemia, hypo- or hypernatremia, hypocalcemia, and hypomagnesemia, should be also discarded. Advanced imaging techniques, such as computed tomography (CT) and magnetic resonance imaging (MRI), can be performed in cases where congenital or acquired abnormalities of the brain and spinal cord are suspected. Other ancillary diagnostic tests, such as cerebrospinal fluid (CSF) collection for culture and cytological evaluation in order to discard meningoencephalitis, could also be performed [52].

The cornerstone of NE treatment is support (nursing or enteral/parenteral nutrition, fluid therapy, management); antimicrobials to prevent infections; and treatment of secondary problems, including oxygen and therapies to ensure correct oxygen delivery and cerebral and organ perfusion (vasopressors, inotropes, etc.); plasma transfusion; seizure control; neuroprotectants; cerebral edema reduction; respiratory stimulants; and antioxidants (Table 7) [52].

Donkey foals can develop tetanus, although not immediately after birth (author experience). Similar to other species, tetanus is associated with wounds being contaminated with *Clostridium tetani*, which produces tetanospasmin. To prevent this condition, it is important that jennies are vaccinated with tetanus toxoid. In addition, hygiene and good care are very important. Tetanus antitoxin can be administered at newborn donkey foals in high-risk regions. Clinical signs are similar to equine foals with tetanus, characterized by increased muscle tone, spasticity, third eyelid prolapse, and even recumbency. These animals usually die from respiratory failure. Treatment is mainly supportive. This is a major cause of foal mortality particularly in developing countries due to the lack of vaccination, the lack of tetanus antitoxin, and scarce veterinary care, although it is also a leading cause in developed countries. The mortality rate can be as high as 66.7% [53].

Botulism can affect donkey foals as early as one week of age (author experience). The main presentation in foals is toxicoinfectious botulism (shaker foal syndrome), where *Clostridium botulinum* proliferates in the gastrointestinal tract and produces botulism toxin. The toxin reaches the neuromuscular junctions and interferes with acetylcholine release, resulting in flaccid paralysis. Prevention can be challenging, because vaccines are not readily available. Treatment is based mainly on supportive care, although botulism antitoxin can be administered in some countries. Mortality is high.

Vitamin E deficiency-related disorders (equine neuroaxonal dystrophy and equine degenerative myeloencephalopathy) have not been reported in this species. In some equine breeds, these disorders may have a genetic basis that worsens with vitamin E deficiency [54]; however, we do not know if donkeys develop vitamin E-associated neurologic disturbances. 

### 3.6. Gastrointestinal Disorders

There are no differences in enteritis, colitis, or colic between donkey and equine foals. Donkey foals can also be born with congenital abnormalities (e.g., atresia coli and ani; Table 8) [45,55].

#### 3.6.1. Colic in Donkey Foals

The causes of abdominal pain in donkey foals are the same as equine foals (Table 8), including gastroduodenal ulcers, enteritis, enterocolitis, meconium retention, and intestinal strangulation.

Donkey foals are stoic, and signs of colic may not be evident, even with strangulating lesions. There is a species-specific pain scale described for adult donkeys [56,57], which could be useful in the diagnosis. Cytologic interpretation of abdominal fluid is similar to equine foals, with similar leukocyte (<3000/µL), total protein (<30 g/L), and lactate (<2 mmol/L) concentrations. The principles of abdominal ultrasonography are the same. Abdominal radiography can be useful for meconium retention diagnosis. Therapeutic principles for medical and surgical colic are similar between species [21]. No outcome information is available on donkey foals with strangulating lesions.

#### 3.6.2. Diarrhea in Donkey Foals

The pathophysiology of enterocolitis seems to be similar to equine foals, with the same pathogenic organisms being involved (Table 8) [35]. Diarrhea can be infectious (viral, bacterial, and parasitic) and non-infectious (caused by lactose intolerance, microbiota changes, parasites, and sand). Predisposing factors are similar to equine foals, with infectious diarrhea being more common in farms with high animal density, no vaccination history, and poor hygienic conditions.

Diagnostic methods for pathogen identification are the same as for equine foals and include serial fecal culture and PCR [58,59,60]. Laboratory findings are nonspecific and include hemoconcentration, leukocytosis or leukopenia, neutrophilia or neutropenia with toxic changes, azotemia, hypoglycemia, hyperlactatemia, as well as acid–base and electrolyte disturbances. Serum IgG concentrations could be low in foals that are a few days old [61,62]. These animals are often septic.

Treatment principles for diarrhea include hemodynamic support, the correction of acid–base and electrolyte abnormalities, and providing energy (parenteral nutrition). Non-steroidal anti-inflammatory drugs (NSAIDs) should be considered to reduce inflammation, pain, and pyrexia and to ameliorate the effects of endotoxemia (Table 5) [63]. However, caution must be taken due to their side effects, particularly in azotemic animals. Antimicrobial selection will depend on clinical presentation, laboratory abnormalities, and bacterial culture results. Newborn foals with gastrointestinal disease often become septic. A broad spectrum of combinations, such as penicillin and amikacin, are frequently used (Table 5). Third-generation cephalosporins may be considered, depending on the stewardship of every country. In foals with enteritis or enterocolitis, administration of metronidazole should be added to the treatment [21]. Metronidazole can cause anorexia [21], which should be taken into consideration in donkeys, in particular, those with increased triglyceride concentrations due to the risk of hyperlipemia. There are pharmacokinetic differences for frequently used antimicrobials not only between donkeys and horses [3] but also between neonates and adults. Pharmacokinetic information for most drugs is lacking for donkey foals. For example, for equine foals, lower doses and longer dosing intervals are recommended for metronidazole [64], but this information is not available for donkey foals. Thus, protocols for equine foals are recommended.

The treatment of parasitic diarrhea will depend on the age of the foal as well as the target parasite and evidence of resistance. Imidazoles (e.g., fenbendazole), in general, are safe, while avermectins (e.g., ivermectin) and milbemycins (e.g., moxidectin) should be avoided in foals of less than 6 months of age, particularly if their bodies are in poor condition, due to the risk of neurologic signs. Praziquantel is suggested for old foals to treat tapeworms. It is recommended to deworm jennies in the last trimester of gestation [65].

Other drugs can also be administered in foals with enterocolitis, such as gastric protectants, proton pump inhibitors, prostaglandin E analogs, and intestinal adsorbents (Table 7). The use of probiotics is controversial. Based on studies in equine foals, probiotics may increase the risk of diarrhea and should be avoided [66]. The microbiota in donkey foals is similar to adult donkeys and horses [67] but different from equine foals [68]. Donkey foals with proximal gastrointestinal disease, whether infectious (*Clostridium* spp., *Salmonella* spp., and rotavirus) or non-infectious (primary intolerance) often develop lactose intolerance, leading to osmotic diarrhea, and will benefit from oral lactase supplementation. Like any animal with suspected infectious diarrhea, biosecurity protocols, including isolation, must be implemented.

Foal heat diarrhea (7–8 days old) [69], antibiotic-induced diarrhea, and diarrhea due to nutritional imbalances may require minimal medical support and have good prognosis.

### 3.7. Respiratory Disorders

Donkey foals develop the same respiratory disorders as equine foals (acquired and congenital) (Table 9), although epidemiological information is lacking. Clinical signs of congenital conditions of the upper respiratory tract are similar to equine foals [70,71], although they could be missed, depending on the type of farm management and geography.

Respiratory diseases can be from respiratory or extra-respiratory causes, infectious or non-infectious (Table 9), and are not different to equine foals. In acquired conditions, clinical signs can be present immediately after birth (similar to congenital disorders) or become evident a few days later. Pneumonia can develop days to weeks later, mainly with fungal pneumonia in immunocompromised foals [72].

Clinical signs are not different from those observed in equine foals, including dyspnea, coughing (poor reflexes in newborn donkeys), milk coming out of the nostrils after nursing, tachypnea, mucopurulent discharge from aspiration pneumonia, and respiratory distress [73]. Cyanotic mucous membranes are observed when PaO_2_ < 65 mmHg, in some cases later in the disease. Like in adult donkeys, lungworms are usually not associated with pulmonary disease in donkey foals, but signs can be observed (chronic cough) in older foals due to roundworm migration.

Diagnostic methods for respiratory disease are the same for equine and donkey foals. These include routine hematology, blood biochemistry, an evaluation of ventilation (blood gases and oximetry), a transtracheal wash, bronchoalveolar lavage, and imaging (endoscopy, ultrasonography, radiography, and tomography). In addition, an evaluation of cardiovascular function is recommended. In older foals, bronchoalveolar lavage may be used to diagnose lungworms [74].

Broad-spectrum antimicrobials, as previously mentioned (Table 5), should be initiated in most sick donkey foals due to respiratory disorders [73], whether the initial cause is infectious or non-infectious. Additional therapeutic approaches include maintaining hydration and tissue perfusion; oxygen delivery; controlling inflammation, fever, and pain (Table 5); and providing energy following similar premises to other disturbances (e.g., parenteral nutrition). Bronchodilators, expectorants, cough suppressants, respiratory stimulants, diuretics, and corticosteroids can be administered to improve ventilation (Table 7).

Similar to equine foals, congenital conditions usually require surgical correction, although aspiration pneumonia from congenital disorders will not be resolved until the primary problem is addressed and secondary infections are controlled.

### 3.8. Musculoskeletal Disorders

Musculoskeletal malformations observed in donkey foals are not different from equine foals and include malformations, digital dysgenesis, patellar luxation, angular and flexural deformities, and brachygnatism [75,76,77].

Acquired musculoskeletal conditions in donkey foals are similar to equine foals, with developmental orthopedic diseases (angular and flexural deformities) being most commonly observed. Management of these disorders will depend on the type of abnormality, duration, size, and age of the foal. Diagnosis can be reached with physical exploration and clinical signs or may require additional methods, including imaging, such as radiography. Due to anatomical and radiographical differences with foals, special considerations must be taken into account for this species [78]. Bandages, intravenous oxytetracycline administration, toe extension, and corrective shoeing may be necessary. Some severe cases may require surgery [79,80].

Similar to equine foals, septic donkey foals are also at risk of developing septic arthritis, physitis, and osteomyelitis, being higher in foals with FTPI [34]. Treatment approaches are similar to equine foals, including antimicrobial administration (intra-articular and regional perfusion) and joint lavage (arthroscopy or through needles).

White muscle disease (WMD) is due to vitamin E and selenium deficiency, which can have different presentations, depending on the severity and affected system. Predisposing factors are arid and acid soils and jennies not being fed good-quality forage or only with a straw [81]. When WMD is suspected, it is recommended to measure selenium or selenium activity via glutathione peroxidase and vitamin E (α-tocopherol) concentrations. Reference values are specific to each laboratory and region. Lower selenium concentrations were reported in mixed-breed healthy donkey foals (0.05 µg/mL) compared to adult donkeys (0.11 µg/mL) in central Italy [82], being also lower compared to horses [83]. In the same study, vitamin E concentrations were similar between donkeys and horses but were lower in donkey foals (5.9 µmol/L) than in adult donkeys (range: 7.7–8.9 µmol/L) [82]. It is important to have age-specific internal reference values in each laboratory. In endemic areas or where WMD has been previously diagnosed, it is important to supplement jennies in the last trimester of pregnancy with oral (daily intake is 0.1–0.15 mg/100 kg) or parenteral (0.03 mg/kg/IM) selenium [84].

Esteatitis and myonecrosis have been described in donkey foals [85,86,87,88], although whether these disorders are due to vitamin E and Se deficiency remains unclear. Pain in the nuchal ligament area is the most common clinical sign, together with others that are nonspecific, such as lethargy, anorexia, subcutaneous edema, fever, and tachycardia. Besides clinical signs, diagnosis is supported by low serum vitamin E and Se concentrations and increased CK, AST, and LDH concentrations [85,86,87,88]. Muscle biopsy could be helpful to assess tissue degeneration, fibrosis, necrosis, inflammatory infiltration, and lipopigment accumulation that is consistent with oxidative damage, as well as in discarding other differential diagnoses [85,86,87,88]. Treatment is based on intramuscular vitamin E and selenium administration followed by oral supplementation (Table 7). Antioxidants and anti-inflammatory drugs are useful for controlling pain and systemic inflammation.

### 3.9. Cardiovascular Disorders

Information on cardiovascular diseases in donkey foals is scarce. Cardiovascular diseases, both congenital and acquired, affecting equine foals can be found in donkey foals.

Some congenital disorders reported in donkey foals include septal defects, tetralogy of Fallot, patent ductus arteriosus, truncus arteriosus, and pseudotruncus arteriosus [89,90].

Dysrhythmias can be detected shortly after birth in donkey foals, but these are usually transient. The principles of electrocardiogram, echocardiography, and thoracic radiography in equine foals are applicable to donkey foals. Antidysrhythmic therapy follows similar premises compared to equine foals [91].

### 3.10. Metabolic and Endocrine Disorders

Hypoglycemia is frequent in sick donkey foals, which often have normal or low insulin concentrations, supporting an appropriate response to energy deprivation [92]. Information on most energy-regulating hormones in donkey foals is minimal.

Hyperlipemia is an important disorder in donkey foals with a negative energy balance, leading to secondary disturbances, such as liver insufficiency, renal dysfunction, dysrhythmias, pancreatitis, and diarrhea, due to fatty tissue infiltration [93].

Like equine foals, most critically ill donkey foals have an appropriate response to stress by releasing adrenocorticotropic hormone (ACTH) and cortisol [94]; however, some can develop relative adrenal insufficiency (RAI), characterized by inappropriately low cortisol concentrations with normal or high ACTH concentrations. This disturbance is known as critical illness-related corticosteroid insufficiency (CIRCI) and can contribute to morbidity and decrease the survival rate [94].

Similar to equine foals, septic neonate donkeys (mostly in those with gastrointestinal disease) can develop hypocalcemia and hypomagnesemia [95], although information on their prevalence or on calcium-regulating hormones is scarce in this species. Critically ill donkey foals with hyperlipemia or those receiving parenteral nutrition may develop hypomagnesemia, hypokalemia, and hypophosphatemia (refeeding syndrome) [95]. Therefore, in addition to calcium treatment, some donkey foals may require intravenous magnesium (magnesium sulfate), phosphorus (sodium phosphate or potassium phosphate), and potassium (potassium chloride or potassium phosphate) supplementation.

### 3.11. Immuno-Mediated Disorders

Although prevalence is unknown in this species, neonatal isoerythrolysis (NI) can occur in donkey foals [96]; however, the prevalence is higher in mule foals (around 10%) [97]. Similarly, the prevalence of IMTP is higher in mule than in donkey foals [98].

The highest incidence of NI in mule foals has been attributed to major antigenic differences between donkeys and horses, in particular to the donkey factor that sensitizes mares to produce antibodies that are passed in colostrum and subsequently to the neonate. In mares that have mule foals, it is recommended to either test them for the presence of anti-donkey antibodies or simply discard their colostrum and provide colostrum to the neonate from a mare that did not foal a mule [99]. After 24 h, the foal can be allowed to nurse from the mare. Information on blood types in donkeys and mules is lacking. The pathogenesis of IMTP is similar to NI, with antibodies in colostrum mediating platelet removal or destruction.

Signs of NI in donkey and mule foals are similar to equine foals [100], ranging from lethargy and anorexia to icteric and pale mucous membranes, tachycardia, and pigmenturia, depending on the colostrum ingested, immunoglobin concentrations, and the degree of hemolysis. No specific signs are observed for IMTP, although petechiae and bleeding can be observed in severe cases.

The diagnosis of NI is similar to that of equine foals [101]. A Coombs test to detect agglutinating antibodies on the surface of erythrocytes could be confirmatory, but a negative test is unlikely to change the therapeutic plan, in particular when there is clinical and laboratory evidence of NI. The diagnosis of IMTP is initially clinical, based on the hemogram (thrombocytopenia) and viscoelastography, but could be confirmed by flow cytometry, which demonstrates antibodies coating the platelets.

Treatment of NI is based on transfusion of washed blood from the jenny. Alternatively, blood from a gelding or a donor that has tested negative for the donkey factor could be considered. Oxygen (5 L/min) or polymerized bovine hemoglobin (5 mL/kg) can be used to improve tissue oxygenation [100]. Platelet-rich plasma transfusion or fresh blood transfusion is recommended for IMTP [100]. In both disorders, steroid administration could be considered. It is important to mention that often donkey foals that receive equine plasma develop IMTP and occasionally IMHA. Therefore, as previously mentioned for colostrum, it is recommended to perform hemograms within 48 h of transfusion.

Other causes of hemolysis in neonates include piroplasmosis and leptospirosis, which depend on the geographical location. Transplacental transmission can occur with both piroplasm parasites (*Babesia caballi* and *Theileria equi*). Colostral antibodies against piroplasmosis from seropositive jennies can decline by 2–3 months of age in donkey foals, being sooner than horse foals (approximately 5–6 months old) [102]. Treatment considerations are similar to adult donkeys [103].

## 4. Conclusions and Further Recommendations

Scarce information on the epidemiology of perinatal diseases is available for donkey foals. The prevalence of these disorders varies per country and is likely higher in regions with financial limitations. It is assumed that the mortality rate is higher in donkey foals compared to equine foals, which could be attributed to poor management, minimal veterinary care, low preventative care, and finances, which is typical of low-income countries. Donkey-specific idiosyncrasies, including their stoic behavior and unique physiology (e.g., higher twin pregnancies rate), also play a role in disease detection and progression and are areas that deserve attention.

Despite the fact that donkey foals suffer from the same disorders as equine foals, with diagnostic approaches and therapeutic options being similar between both species, pharmacokinetic data are not available for most used drugs, and doses and interval dosing protocols must be extrapolated from equine foals with caution.

## Figures and Tables

**Table 1:** Modified Apgar score for donkey foals [10,16].

	Score		
Parameter	0 Points	1 Points	2 Points
Heart rate/pulse quality	Undetectable	<60 beats/min, irregular	>60 beats/min, regular
Respiratory rate/pattern	Undetectable	Slow/irregular	40–60 breaths/min, regular
Muscle tone	Limp/absent	Lateral recumbency, some tone, some flexion of limbs	Sternal recumbency
Nasal stimulation	Unresponsive	Grimace, mild rejection	Cough or sneeze
Mucous membranes	Cyanotic	Pale/Pink	Pink

**Table 2:** Hematology of healthy donkey foals.

Age	RBC (×10^6^/µL)	Hb (g/dL)	Hct (%)	WBC (×10^3^/µL)	Neu (×10^3^/µL)	Lym (×10^3^/µL)	Mono (×10^3^/µL)	Eos (×10^3^/µL)	Baso (×10^3^/µL)	PLT (×10^3^/µL)
***Birth***	9.8	17.4	48.2	6.5	5.2	1.4	0.10	0.09	0.08	288
***24 h***	8.8	15.3	41.8	7.3	4.9	2.0	0.13	0.07	0.05	280
***48 h***	8.1	14.5	39.8	8.1	5.9	1.8	0.22	0.07	0.04	288

Data are expressed as means. Baso, basophils; Eos, eosinophils; Hb, hemoglobin; Hct, hematocrit; Lym, lymphocytes; Mono, monocytes; Neu, neutrophils; PLT, platelet count; RBC, red blood cell count; WBC, white blood cell count. Modified from [19,20,21].

**Table 3:** Biochemistry of healthy donkey foals.

Age	Glucose (mg/dL)	BUN (mg/dL)	Creatinine (mg/dL)	TP (g/dL)	Albumin (g/dL)	TBil (mg/dL)	AST (IU/L)	GGT (IU/L)	CK (IU/L)	LDH (IU/L)	ALP (IU/L)	Lactate (mmol/L)	TGL (mg/dL)
***Birth***	111	36.1	2.0	4.4	3.1	0.3	116	23.7	103	284	972	5.5	35
***24 h***	130	33.3	1.4	4.8	3.0	0.3	222	36.7	64	379	1015	1.5	46
***48 h***	134	26.5	1.2	5.0	3.1	0.3	250	34.1	70	348	745	1.6	58

Data are expressed as means. ALP, alkaline phosphatase; AST, aspartate aminotransferase; CK, creatine kinase; GGT, gamma glutamyl transferase; LDH, lactate dehydrogenase; TBil, total bilirrubin; TGL, triglycerides; TP, total protein. Modified from [19,20,21,23].

**Table 4:** Electrolytes of healthy donkey foals.

Age	Sodium (mEq/L)	Chloride (mEq/L)	Potassium (mEq/L)	Total Calcium (mg/dL)	Total Magnesium (mg/dL)	Phosphorus (mg/dL)
***Birth***	135	101	4.4	11.4	1.8	4.4
***24 h***	136	136	4.2	11.2	1.9	4.9
***48 h***	135	135	4.5	11.4	1.9	6.1

Data are expressed as means. Modified from [19,20,21].

**Table 5:** Antimicrobials and anti-inflmatory drugs used in donkey foals for body system disorders.

Drug	Dose	Route	Interval
***Antimicrobials***			
Amikacin	15–25 mg/kg	IV	24 h
	125–1000 mg	IVRLP	24 h
Ampicillin sodium	15–50 mg/kg	IV, IM	8–12 h
Azithromycin	10 mg/kg	PO	24 h for 5 days, then every 48 h
Cefepime ^¶^	11 mg/kg	IV	8 h
Cefotaxime ^¶^	40–50 mg/kg	IV	6 h
Cefpodoxime ^¶^	10 mg/kg	PO	6–8–12 h
Cefquinome ^¶^	1–2.5 mg/kg	IV, IM	6–12 h
Ceftazidime ^¶^	40–50 mg/kg	IV	6 h
Ceftiofur sodium ^¶^	2–10 mg/kg	IV, IM	6–12 h
Ceftriaxone ^¶^	25 mg/kg	IV	12 h
Clarithromycin	7.5 mg/kg	PO	12 h
Chloramphenicol	50–60 mg/kg	IV, PO	6 h
Doxycycline	10 mg/kg	PO	12 h
Gentamicin	10–12 mg/kg	IV	12–24 h
Imipenem and cilastatin ^¶^	10–20 mg/kg	IV	6–12 h
Metronidazole	10–15 mg/kg	IV, PO	8–12 h
Oxytetracycline	5–7 mg/kg	IV	12 h (*Lawsonia intracellularis*, *Anaplasma phagocytophilum*). Higher doses are used for contracted tendons.
Penicillin K or Na	22,000–44,000 IU/kg	IV	6 h
Penicillin procaine	22,000–44,000 IU/kg	IM	12–24 h
Rifampin ^¶^	5–10 mg/kg	PO	12–24 h
Sulfonamide/trimethoprim	20–30 mg/kg	PO	12 h
***Non-steroidal and steroidal anti-inflammatory***			
Acetaminophen (paracetamol)	10–30 mg/kg	IV, PO	12–24 h
Carprofen	0.5–0.7 mg/kg	PO	24 h
Dexamethasone	0.05–0.2 mg/kg	IV, IM, PO	12–24 h
Diphenhydramine hydrochloride	0.5–2 mg/kg	IV, IM	
Dipyrone (metamizole)	10–22 mg/kg	IV, IM	8–12 h
Firocoxib	0.1 mg/kg	IV	12–24 h
Flunixin meglumine	0.25–1.1 mg/kg	IV	8–12 h
Ketoprofen	1.1–2.2 mg/kg	IV	12–24 h
Meloxicam	0.6 mg/kg	IV	12–24 h
Prednisolone	0.5–2 mg/kg	PO	24 h

IM, intramuscular; IV, intravenous; IVRLP, intravenous regional limb perfusion; PO, per os, orally. Most of these drug doses were extrapolated from equine foals or the authors’ experience. Some of these drugs may not be available, depending on the country. ^¶^ Consider country antimicrobial stewardship and legislation. Modified from [21].

**Table 6:** Differential diagnosis for neurologic clinical signs in neonate and older donkey foals.

Nervous System Causes		Extra-Nervous System Causes	
Congenital	Acquired	Congenital	Acquired
- Cerebellar abiotrophy - Dandy–Walker malformation - Deafness - Epidermoid cyst - Epilepsia (juvenile and idiopathic) - Hydrocephalus/Hydranencephaly - Myelodysplasia - Narcolepsy - Syringomyelia	- Bacterial meningoencephalomyelitis: *E. coli*, *Klebsiella* spp., *Actinobacillus* spp., *Salmonella* spp., *Streptococcus* spp., and *Rhodococcus* equi - Fungal meningoencephalomyelitis - Neonatal encephalopathy (NE/NMS/PAS/HIE) - Prematurity/Dysmaturity - Trauma (brain and spinal cord)	- Meningocele/Meningoencephalocele/Meningomyelocele - Occipitoatlantoaxial malformation - Spina bifida - Vertebral body malformation (hypoplasia, fusion, hemivertebrae, etc.) - Vertebral column malformation (lordosis, kyphosis, and scoliosis) - Vertebral stenotic myelopathy (Wobbler syndrome, etc.)	- Acid–base imbalances - Botulism - Electrolyte imbalances (hyponatremia, hypernatremia, hypocalcemia, and hypomagnesemia) - Hypoglycemia - Iatrogenic - Idiopathic epilepsy - Kernicterus (isoerythrolysis, piroplasmosis, liver disease, etc.) - Peripheral nerve paralysis (dystocia, trauma, and NMS) - Poisoning (pesticides, herbicides, etc.) - Prematurity/Dysmaturity - Tetanus - Toxicity (ivermectin, moxidectin, domperidone, levamisole, lidocaine, etc.) - Trauma - Vertebral stenotic myelopathy - Volume overload (cerebral edema) - White muscle disease (selenium deficiency)

HIE, hypoxic-ischemic encephalopathy; NE, neonatal encephalopathy; NMS, neonatal maladjustment syndrome; PAS, perinatal asphyxia syndrome. Many of these disorders have not been described in donkeys in the literature, but they can also occur. Modified from [21].

**Table 7:** Drugs used in donkey foals for body system disorders.

Drug	Dose	Route	Interval
***Gastric acid reducers and protectants, and absorbent***			
Activated charcoal	CJ	PO	CJ
Bismuth subsalicylate	CJ	PO	CJ
Famotidine	2–4 mg/kg	PO	8–12 h
	0.5–1 mg/kg	IV	8–12
Kaolin	CJ	PO	CJ
Lactase	2000–4000 IU/foal	PO	4–6 h
Misoprostol	1–4 µg/kg	PO	8–24 h
Omeprazole	0.5 mg/kg	IV	24 h
	1–2 mg/kg	PO (prevention)	24 h
	2–4 mg/kg	PO (treatment)	24 h
Pantoprazole	1–1.5 mg/kg	IV, PO	24 h
Psyllium	CJ	PO	CJ
Ranitidine	0.9–1.5 mg/kg	IV, IM, PO	6–8 h
Smectite	CJ	PO	CJ
Sucralfate	20–40 mg/kg	PO	6–8 h
***Respiratory tract***			
Caffeine	10 mg/kg	PO (loading dose)	12 h
	2–3 mg/kg	PO (maintenance)	
Clenbuterol	0.5–5 μg/kg	PO, IV, INH	12 h
Doxapram hydrochloride	0.5 mg/kg	IV	As needed
Fluticasone	100–500 μg/50 kg	INH	12–24 h
Furosemide	0.5–2 mg/kg	IV, CRI	4–6 h
Oxygen	5–10 L/min	INH	CJ
***Neurologic and musculoskeletal***			
Dimethyl sulfoxide	1 g/kg as a solution <10%	IV	12–24 h (<10% solution, slowly)
Magnesium sulfate	20–50 mg/kg	IV (loading dose)	CJ
	10–25 mg/kg/h	CRI	
Mannitol	0.25–0.5 g/kg over 30 min	IV (oliguria)	Use as 20% solution, slowly
	0.5–2 g/kg over 30–60 min	IV (cranial/spinal trauma)	
Selenium	1–2 mg	IM	Once
Thiamine	1–10 mg/kg	IV, IM	12–24 h
Vitamin C	30–50 mg/kg	IV	12–24 h
Vitamin E	6.6 IU/kg	IM	24 h for 3 days
	10–20 IU/kg	PO	24 h
***Anticonvulsants, sedatives, and tranquilizers***			
Acepromazine	0.02–0.1 mg/kg	IV, IM	
Butorphanol	0.02–0.1 mg/kg	IV, IM	
Detomidine	0.02–0.04 mg/kg	IV, IM	
Diazepam	0.02–0.1 mg/kg	IV	CJ
Flumazenil	0.01–0.02 mg/kg	IV slowly	
Ketamine	0.5–1 mg/kg	IV (loading dose)	
	0.4–0.8 mg/kg/h	CRI	
Midazolam	0.02–0.4 mg/kg	IV	CJ
Phenobarbital	2–10 mg/kg	IV	8–12 h. Use when foal is no responsive to other drugs
Romifidine	0.05–0.1 mg/kg	IV, IM	
Xylazine	0.5–1.1 mg/kg	IV	
	1–2 mg/kg	IM	

CJ, clinician judgment or manufacturer recommendation; CRI, continuous rate infusion; IM, intramuscular; INH, inhaled; IV, intravenous; PO, per os, orally. Most of these drug doses were extrapolated from equine foals or the authors’ experience. Some of these drugs may not be available, depending on the country. Modified from [21].

**Table 8:** Differential diagnosis of diarrhea and colic in donkey foals.

Diarrhea		Colic	
Non-Infectious Causes	Parasites and Infectious Causes	Strangulating Causes	Non-Strangulating Causes
- Antibiotic-induced diarrhea - Foal heat - Foreign bodies (shaving, wire, etc.) - Lactose intolerance - Microbiota imbalances - Necrotizing enterocolitis - Nutritional dysbiosis - Pancreatitis - Peritonitis - Sand accumulation/Pica - Uroperitoneum	Bacterial: - *Bacteroides* spp. - *Clostridioides difficile* - *Clostridium perfringens* - *Enterococcus durans* - *E. coli* - *Lawsonia intracellularis* - *Rhodococcus equi* - *Salmonella* spp. Viral: - Coronavirus - Rotavirus Protozoal and parasitic: - *Cryptosporidium parvum* - *Giardia* spp. - *Parascaris equorum* - *Strongyloides westeri*	- Colon displacement/torsion - Congenital causes: Meckel’s diverticulum and mesodiverticular band - Hernia: diaphragmatic, inguinal (scrotal), and umbilical - Intussusception (small and large intestine) - Small and large intestine volvulus	- Congenital abnormalities: atresia ani and coli - Endoparasitism (*Parascaris equorum* impaction) - Enterocolitis - Foreign bodies (shaving, etc.) - Gastroduodenal ulcers and duodenal stenosis - Ileus - Lactose intolerance - Meconium impaction - Overo white lethal syndrome (ileocolonic aganglionosis) - Peritonitis - Rectal prolapse - Rib fracture - Uroperitoneum

Many of these disorders have not been described in donkeys in the literature, but they can occur. Modified from [21].

**Table 9:** Differential diagnoses for respiratory diseases in donkey foals.

Respiratory Causes		Extra-Respiratory Causes	
Congenital	Acquired	Congenital	Acquired
- Choanal atresia - Cleft palate - Epiglottis hypoplasia/cyst - Laryngeal deformity - Lung immaturity - Guttural pouch tympany - Pulmonary hypoplasia - Rostral displacement of palatopharyngeal arch - Nares stenosis - Sinus cyst - Soft palate displacement - Surfactant deficiency - Tracheal collapse - Wry noise	- Pneumonia: *Bacterial:* Neonate (*Streptococcus* spp., *Actinobacillus* spp., *Klebsiella* spp., and *Actinobacillus* spp.). Older foals (*Rhodococcus equi*, *Streptococcus equi equi*, and *Streptococcus equi zooepidemicus*). *Viral*: EHV-1, EHV-4, EVA, and equine influenza virus *Fungal*: *Aspergillus fumigatus*, *Cryptococcus neoformans*, *Coccidioides immitis*, and *Pneumocystis carinii* *Parasitic*: *Parascaris equorum*, *Strongyloides westeri*, *Strongylus vulgaris*, and *Dictyocaulus arnfieldi* - Acute respiratory distress syndrome - Foreign bodies - Guttural pouch tympany/empyema - Meconium or milk aspiration - Pneumothorax - Pulmonary edema - Secondary surfactant deficiency due to sepsis, pneumonia, edema, etc. - Tracheal rupture/collapse - Trauma	- Cardiac disease - Diaphragmatic hernia - Neurologic disorders - Rib deformity	- Abdominal distension - Acid–base imbalances - Acute kidney injury (anuria and oliguric) - Anemia - Botulism - Cardiac disease - Diaphragmatic hernia - Dysphagia - Fever - Meningoencephalitis - Neonatal encephalopathy - Pain - Pulmonary hypertension - Rib fractures (pneumothorax) - Trauma - Tetanus - Transient tachypnea - Volume overload - White muscle disease (selenium deficiency)

EHV, equine herpesvirus; EVA, equine viral arteritis. Many of these disorders have not been described in donkeys in the literature, but potentially, they can occur. Modified from [21].

## Data Availability

No new data were created or analyzed in this study. Data sharing is not applicable to this article.
